# Fine-Scale Variation and Genetic Determinants of Alternative Splicing across Individuals

**DOI:** 10.1371/journal.pgen.1000766

**Published:** 2009-12-11

**Authors:** Jasmin Coulombe-Huntington, Kevin C. L. Lam, Christel Dias, Jacek Majewski

**Affiliations:** 1 Department of Human Genetics, McGill University, Montreal, Québec, Canada; 2 McGill University and Génome Québec Innovation Centre, Montréal, Québec, Canada; University of Geneva Medical School, Switzerland

## Abstract

Recently, thanks to the increasing throughput of new technologies, we have begun to explore the full extent of alternative pre–mRNA splicing (AS) in the human transcriptome. This is unveiling a vast layer of complexity in isoform-level expression differences between individuals. We used previously published splicing sensitive microarray data from lymphoblastoid cell lines to conduct an in-depth analysis on splicing efficiency of known and predicted exons. By combining publicly available AS annotation with a novel algorithm designed to search for AS, we show that many real AS events can be detected within the usually unexploited, speculative majority of the array and at significance levels much below standard multiple-testing thresholds, demonstrating that the extent of cis-regulated differential splicing between individuals is potentially far greater than previously reported. Specifically, many genes show subtle but significant genetically controlled differences in splice-site usage. PCR validation shows that 42 out of 58 (72%) candidate gene regions undergo detectable AS, amounting to the largest scale validation of isoform eQTLs to date. Targeted sequencing revealed a likely causative SNP in most validated cases. In all 17 incidences where a SNP affected a splice-site region, *in silico* splice-site strength modeling correctly predicted the direction of the micro-array and PCR results. In 13 other cases, we identified likely causative SNPs disrupting predicted splicing enhancers. Using Fst and REHH analysis, we uncovered significant evidence that 2 putative causative SNPs have undergone recent positive selection. We verified the effect of five SNPs using *in vivo* minigene assays. This study shows that splicing differences between individuals, including quantitative differences in isoform ratios, are frequent in human populations and that causative SNPs can be identified using *in silico* predictions. Several cases affected disease-relevant genes and it is likely some of these differences are involved in phenotypic diversity and susceptibility to complex diseases.

## Author Summary

Alternative splicing (AS), through the alternative use of exons, can produce many different mRNA transcripts from the same genomic locus, thus possibly resulting in the production of many different proteins. We know that splicing differences between individuals exist and that these changes are often associated with genetic variants. Thus far, very few of these associations have led to the precise localization of the causative polymorphisms. In this work, using in-depth analysis of previously published splicing sensitive micro-array data from human cell lines, we identified and validated a large number of splicing changes which are highly correlated with nearby genetic variations. We then sequenced the genomic DNA around candidate exons and used *in silico* modeling tools to identify causative SNPs for most of our candidates. Using a plasmid reporter construct, we further demonstrated that five selected SNPs reproduce the expected effect *in vivo*. Our results indicate that genetically controlled splicing differences between individuals may be more common than previously suggested and can be very subtle; and most are caused by SNPs affecting either the splice-site region or exonic splicing enhancers (ESEs) sequences.

## Introduction

Alternative splicing (AS) allows for multiple mRNA isoforms to be transcribed from a single gene locus, potentially creating much greater protein diversity from our roughly 25 thousand human genes [1]. AS is very common in higher order organisms and, especially through the lens of newer, high throughput technologies, such as oligonucleotide arrays and transcriptome sequencing, we are finally realizing the true extent and importance of AS. New studies based on deep sequencing and micro-arrays with exon junction probes estimate the proportion of genes undergoing AS in humans between 74% and 94% [2]–[5] Hence, it is crucial to understand the functions and the regulation of AS if we are to arrive at a real grasp of regulation of gene expression and gene networks. Although most isoform variation is thought to occur between tissues, many differences exist among healthy individuals in a population [6]–[8]. It is likely these differences are of genetic origin and contribute to phenotypic diversity and disease susceptibility. Many Mendelian disorders, such as cystic fibrosis [9], have been explained by splicing errors caused by genetic mutations [10]. This shows the importance of finding more genetically driven isoform variations to understand the genetic causes of complex diseases.

Until recently, AS differences were not detectable with commercially available micro-array platforms. Due to low probe densities, those platforms only aimed at measuring gene-level expression and targeted mainly the 3′ untranslated region (UTR) of genes. The Affymetrix Exon Array, with its nearly 5.5 million exon-targeted probes, is one of the recent genomic tools available for profiling of splicing or transcript initiation/termination differences between human tissues or between individuals.

In the prelude to this work, Kwan et al. [6] showed using Exon-Array expression data from lymphoblast cell-lines of HapMap individuals that cis-acting polymorphisms are associated with many gene-level expression differences and isoform ratio differences between individuals of the HapMap CEPH population [11]. While the initial analysis detected numerous robust differential splicing events that were genetically controlled, the study did not attempt to identify the actual causal polymorphisms and it was not clear whether it had sufficient statistical power and signal to noise ratio to detect more subtle genetic influences on exon inclusion levels. Given the high validation rate achieved for AS in the first study, we expected to find many more inter-individual splicing differences deeper, within the statistically less significant candidates and also within the speculative content of the microarray, which targets predicted or rarely expressed exonic regions. Moreover, we aimed to determine whether it was possible to identify the causative polymorphisms – as opposed to extended regulatory haplotypes – responsible for such changes. Thus, in order to increase our detection threshold, in the analysis presented here we made use of publicly available, prior AS information. Using a customized heuristic optimized for the detection of AS events and some visual curation, we selected a large sample of candidate genetically regulated AS events. Subsequently, we validated these events using RT-PCR, and in order to identify likely causative SNPs, we re-sequenced the genomic DNA around the alternatively spliced regions. Finally, we used *in silico* predictions and, in selected cases, minigene assays, to verify the causative nature of the detected polymorphisms. This new analysis on the data demonstrates that the Exon Array can detect much more subtle splicing differences than initially suggested and that the speculative, non-core probesets, which constitute the majority of the array, contain useful data for AS discovery. This study constitutes the most in-depth analysis of cis-regulatory heritable splicing differences to date, having validated quantitatively the greatest number of cases and found a likely causative SNP is most cases. Our results provide new insights in the genetic regulation of splicing, demonstrate that subtle differences in alternative splicing between humans are more frequent than initially detected, and that the causative polymorphisms can be identified and validated in reporter mini-gene systems.

## Results

### Selecting candidate AS events

To optimize our chances of finding true AS events, we only considered candidate probesets whose target genomic coordinates overlapped an AS event catalogued in at least one database, including KnownAlt [12], ASAP-II [13] and additional events inferred directly from EST/mRNA genome annotations (see Methods). We ranked candidates statistically according to both the regression p-value and a custom-designed measure representing the unexpectedness of the probeset's fold-change in the context of the other probesets in the gene (see Methods). Further, by visually inspecting probeset fold-changes and regression P-values of the top candidate genes in the UCSC Genome Browser, we selected 68 new potential AS events, along with 4 events from previous analyses [6], for further validation and characterization. For the purpose of this analysis, we chose events that would be easily amplifiable via PCR, therefore excluding large intron retentions or alternative transcript initiation/termination events. The UCSC Genome Browser Tracks of selected candidates are available in Table S1. The red “AS-marker” track indicates the position of the affected exon.

### PCR validation

**Figure 1:**
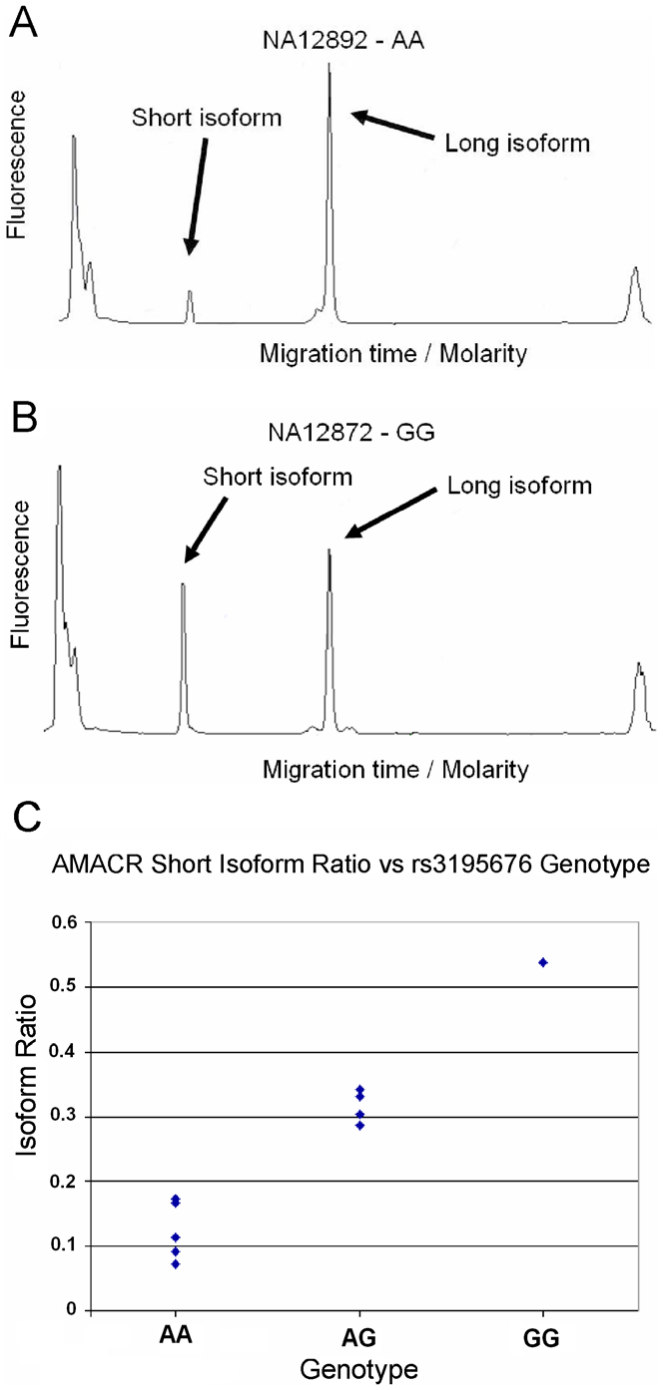
Validating isoform eQTLs from automated capillary electrophoresis of RT–PCR products. (A,B) Example capillary electrophoresis fluorescence readings for the AMACR gene for individuals with AA and GG genotypes for SNP rs3195676. (C) Estimated isoform ratios of the AMACR gene for each individual, as a function of the SNP Genotype. See Methods for details.

In order to detect isoform ratio differences quantitatively as well as qualitatively, we validated our candidate events using semi-quantitative RT-PCR. Instead of performing the validations on all 57 individuals, we chose a strategic sub-sample of 10 individuals from the HapMap CEPH population, which was the smallest possible sample for which at least one individual was polymorphic at every SNP associated with an AS event. The primers were targeted to the two exons flanking each alternatively spliced region. Product abundance was quantified as described in the Methods. Figure 1A and 1B show examples of electrophoresis readings from which the isoform ratios were estimated. Out of the 58 candidates which produced interpretable results, 22 showed a significant association with the SNP (p<0.05, Figure 1B shows an example), confirming that the AS event is under the control of a cis-regulatory mutation, 10 showed a visible trend in the expected direction, 7 showed clear differences in isoform ratios between individuals but with no obvious trend linked to the SNP genotype, and 3 showed visible isoforms with no detectable differences in ratios. The remaining 16 showed no evidence of AS. It should be noted that the small sample size (10 individuals) and limited representation of different genotypes limits the power of this validation approach. Thus, while observing a single PCR product in all samples can be considered as a reliable indication of a false positive result, observing two alternatively spliced products of the expected sizes, even without achieving statistical significance, is evidence supporting the initial microarray finding. Thus depending on the stringency of the validation criteria – detecting a statistically significant association within the PCR data, versus observing two alternatively spliced products - our validation rate is between 38% and 72%. Table 1 shows all validated AS events organized by the strength of the validation evidence.

**Table 1:** RT–PCR validated AS candidates.

Gene	Probeset ID	Gene region	AS Type	Most likely Causative SNP^1^	Array regression P-value	PCR validation regression p-val^2^
C14orf129	3550335	5′ UTR	Alternative SS	rs2053588	3.40E-06	1.90E-09
C8orf59	3142947	5′ UTR	Cassette exon	chr8:86318715	3.10E-27	1.30E-06
AMACR	2852757	Coding	Cassette exon	rs10941112	2.40E-08	2.70E-06
USP8	3593685	Coding	Cassette exon	rs4775889	3.40E-06	7.10E-06
PLD2	3707250	Coding	Alternative SS	rs3764897	1.80E-06	5.30E-06
ZNF419	3843285	Coding	Cassette exon	rs11672136	3.00E-07	7.10E-06
UEVLD	3365492	Coding	Cassette exon	rs56151250	2.70E-08	2.30E-05
SMAD5	2830027	5′ UTR	Cassette exon	unknown	2.00E-05	1.60E-04
TMEM77	2427753	5′ UTR	Cassette exon	rs3762374	2.70E-15	1.70E-04
SERGEF	3365169	Coding	Cassette exon	rs211146	1.20E-10	1.90E-04
MMAB	3470844	Coding	Cassette exon	rs2287180	4.20E-08	8.00E-04
SNORD49	3712109	Coding	Cassette exon	unknown	5.70E-07	9.50E-04
RNASEN	2852054	Coding	Cassette exon	rs55656741	4.50E-06	4.30E-03
DUSP18	3957502	5′ UTR	Cassette exon	rs5753268	1.70E-10	6.30E-03
IFI44L	2343481	Coding	Cassette exon	rs1333973	1.40E-06	7.80E-03
SH3YL1	2537134	Coding	Cassette exon	rs62114506	1.10E-09	1.10E-02
SLC3A2	3333716	Coding	Cassette exon	unknown	1.40E-11	1.10E-02
WDR67	3114099	Coding	Cassette exon	rs6984928	1.30E-08	1.40E-02
WARS	3579582	5′ UTR	Cassette exon	rs941928	1.10E-13	1.50E-02
TMEM149	3859924	Coding	Alternative SS	rs17638853	7.90E-07	1.60E-02
DMKN	3859789	Coding	Cassette exon	rs4254439	2.00E-06	2.20E-02
RBCK1	3873192	Coding	Cassette exon	rs41281892	1.00E-07	2.50E-02
ESPL1	3415861	Coding	Alternative SS	rs6580942	3.10E-09	correct trend
RGL3	3850922	Coding	Cassette exon	unknown	2.80E-06	correct trend
XPNPEP3	3946515	Coding	Cassette exon	unknown	7.80E-09	correct trend
SGOL1	2665585	Coding	Cassette exon	rs61729306	1.70E-10	correct trend
GTF3C2	2545738	5′ UTR	Cassette exon	unknown	3.10E-07	correct trend
PPIL2	3938300	3′ UTR	retained intron	rs12484060	1.00E-10	correct trend
ACP1	2466156	Coding	Cassette exon	rs11553746	2.70E-12	correct trend
MGC16169	2780811	Coding	Cassette exon	rs12639869	2.90E-17	correct trend
CCDC41	3466174	5′ UTR	Cassette exon	chr12:93353207	1.70E-05	correct trend
DDX19A/B	3667169	Coding	Cassette exon	unknown	7.30E-04	correct trend
HNRPH1	2890160	Coding	retained intron	rs34734159	4.10E-13	variable ratios
BCKDHA	3834195	Coding	Alternative SS	rs12602	9.20E-05	variable ratios
FAM64A	3707965	3′ UTR	retained intron	rs7218283	3.40E-13	variable ratios
ZNF83	3869658	Coding	retained intron	rs7248435	2.70E-10	variable ratios
IKIP	3467329	Coding	Cassette exon	unknown	2.40E-05	variable ratios
SIDT1	2636499	Coding	Cassette exon	rs2271494	6.70E-04	variable ratios
IL6	2992594	Coding	Cassette exon	rs2069832	8.10E-05	variable ratios
VISA	3874507	Coding	Alternative SS	rs17857295	1.10E-12	isoforms detected
UBAP2	3203812	Coding	Cassette exon	rs307682	1.30E-07	isoforms detected
USP36	3772596	3′ UTR	retained intron	unknown	2.20E-07	isoforms detected

1 Coordinates are given when SNP does not exist in dnSNP. “Unknown” indicates there was no sequence information or very poor quality sequencing results.

2 P-value of the most significant correlation between an isoform's ratio and the associated SNP genotype.

### Identifying causative polymorphisms

For all the candidates selected in this study and a few additional validated candidates from the previous analysis [6], we sequenced a region of 600–800 bps around the putative AS events in 2 individuals predicted to preferentially express one isoform and 2 individuals expressing the other. The selection of individuals was based on both the genotype of the associated SNP and the micro-array expression scores for the probeset of interest. Thus, even if the associated SNP was not perfectly linked to the causative SNP, selecting for extreme expression phenotypes increased the chances that the 4 individuals would differ at the causative polymorphism. Analysis of the sequencing results revealed 86 polymorphisms in 60 confidently sequenced regions, 76 of which were already catalogued in dbSNP129 [14], and 10 which were novel. Out of the 34 validated AS events which were successfully sequenced (including 4 from the previous analysis), all showed at least one SNP within the sequenced interval, as did 6 out of the 7 sequenced regions which were negatively validated. For each gene in which one or more SNPs were identified, Table S2 links to custom UCSC Genome Browser tracks which indicate the position of SNPs and the associated fold-change in sequenced individuals.

**Figure 2:**
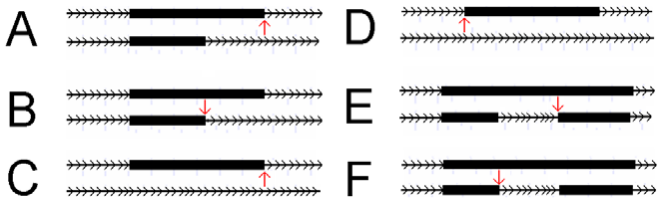
AS type and affected splice-site for SNPs identified in Table 2 and Table 3. The arrow indicates the splice-site affected by the polymorphism. The genes are read from left to right, as indicated by the intersecting arrow heads. The type of AS event and which splice-site is affected is essential to understanding the relation between the probeset expression change and the theoretical efficiency of splicing. In (A,C,D), the correlation should be positive since the use of the splice-site produces a longer transcript, while in (B,E,F), an inverse relation is expected since the use of the splice-site produces a shorter transcript.

For 24 of the genes, we could identify a SNP within 20 bases of a relevant splice-site. Using the MaxEntScan algorithm [15], which calculates the theoretical strength of splice-sites based on maximum entropy, we could verify whether the expected effects of many SNPs adequately explained the difference in micro-array expression between the sequenced individuals. In all 17 cases where differences in splice site strength could be calculated, the micro-array results and the maximum entropy scores of the polymorphic splice-site sequences agreed on the direction of the effect (See Table 2). Figure 2 shows the types of AS events and the relative position of the affected splice-site, which is crucial to understanding the direction of the expression changes. For an additional 16 cases where a SNP was present within the affected exon, we used the ESE Finder 3.0 online tool [16] to predict ESEs affected by the SNP and assign them a matrix-based affinity score (see Table 3). In 13 out of the 16 exons, the predicted change in the number and/or affinity of ESE motifs was concordant with the probeset expression change. Although, in some of these cases, identifying the affected splice-site is not obvious, we are still able in all cases to infer the direction of the predicted probeset expression change, as shown in Figure 2. The 3 for which the predicted effect was in disagreement were the 3 cases with the smallest expression fold-change. This result is encouraging, particularly since it only concerns the binding preferences of 4 splicing factors, out of possibly dozens, and the less than perfect agreement between predictions and results likely reflects the relative lack of detailed understanding of ESE motifs as compared to splice-site consensus sequences.

**Table 2:** SNPs affecting splice-sites.

Gene	SNP ID/new SNP	AS Type^1^	Splice-site sequence^2^	Maximum Entropy Score^3^	Probeset Expression^4^
C8orf59	new SNP	A	aagG**T**aaaa	8.38	138
			aagG**A**aaaa	0.19	12
DMKN	rs4254439	C	**c**ggGTgagc 5	8.18	117
			**a**ggGTgagc	7.75	11
ERAP2	rs2248374	B^6^	atgGT**a**agg 5	9.33	69
			atgGT**g**agg	7.61	297
MGC16169	rs12639869	C	aa**g**GTatgt 5	9.79	225
			aa**t**GTatgt	5.87	26
PLD2	rs3764897	A	c**a**gGTagag 5	7.10	140
			c**g**gGTagag	2.04	43
SH3YL1	rs62114506	C	atgGTaa**g**t 5	11.01	118
			atgGTaa**c**t	6.06	22
TMEM77	rs3762374	C	gttGTga**g**t 5	6.59	2552
			gttGTga**a**t	−4.72	394
ZNF419	rs11672136	D	cca**t**AGgtt 5	8.87	56
			cca**a**AGgtt	6.65	13
PARP2	rs2297616	A	ctgGTa**a**ga	9.45	1378
			ctgGTa**g**ga 5	6.77	86
ULK4	rs1716698	C	aagGT**a**ggg 5	8.76	123
			aagGT**c**ggg	3.63	13
FAM64A	rs7218283	E^6^	ttg**c**AGgaa 5	5.25	12
			ttg**a**AGgaa	1.79	73
IFI44L	rs1333973	C	aagGT**a**tgt 5	9.79	4602
			aagGT**t**tgt	7.81	711
PPIL2	rs12484060	B^6^	**c**agGTtggc 5	5.52	154
			**t**agGTtggc	2.04	370
OVGP1	rs1264894	D	**a**cacAGggc	10.03	101
			**g**cacAGggc 5	9.22	28
TMEM149	rs17638853	A	cag**G**Tgagc	9.60	193
			cag**A**Tgagc 5	1.42	32
C14orf129	rs2053588	B^6^	ca**g**GTactg	9.04	9
			ca**a**GTactg 5	0.23	62
CAST	rs7724759	C	tc**g**GTgagt 5	11.11	647
			tc**a**GTgagt	7.68	117

1 See Figure 2 for a graphical depiction of the two alternative isoform structures and the relative postion of the SNP-affected splice-site.

2 Upper-case bases represent consensus donor/acceptor site and bold font indicates SNP.

3 Maximum entropy score as calculated using MaxEntSCan [15].

4 Averaged PLIER-summarized expression score for each homozygous genotype.

5 Ancestral genotype, as inferred from the chimpanzee genome.

6 Cases for which an inverse correlation between splice-site strength and probeset expression is expected based on the two isoform structures and the position of the affected splice-site, as shown in Figure 2.

**Table 3:** SNPs affecting predicted exonic splicing enhancers (ESEs).

Gene	SNP ID	AS Type^1^	Allele	Splicing Enhancer Sequence^2^	Splicing Factor	ESE Finder Score^3^	Probeset expression^4^
VISA	rs17857295	C	C	CTAC**C**AG	SRp40	2.80	429
				C**C**AGAGC	SRp40	2.80	
				GTCTAC**C**A	SC35	4.01	
				**C**AGAGCT	SF2/ASF^6^	2.94	
			G^5^	None	-	-	17
SGOL1	rs61729306	C/D	A	CAC**A**CTG	SF2/ASF^6^	3.42	95
				C**A**CTGGG	SF2/ASF^6^	3.10	
				AC**A**CTGG	SRp40	5.03	
			T^5^	C**T**CTGGG	SF2/ASF^6^	2.67	5
				AC**T**CTGG	SRp40	3.98	
WARS	rs941928	D	C	GTGTA**C**TA	SC35	2.34	184
			G^5^	None	-	-	12
UEVLD	rs56151250	C	G	**G**CATTCTG	SC35	2.41	52
				T**G**CATT	SRp55	2.97	
			C^5^	None	-	-	5
AMACR	rs10941112	C	A	TG**A**CAAG	SRp40	4.94	381
				CTG**A**CAA	SF2/ASF^6^	2.81	
			G^5^	GCACTG**G**	SRp40	3.57	76
DUSP18	rs5753268	C	T	AACC**T**CTA	SC35	3.13	58
				CC**T**CTAC	SRp40	3.26	
			C^5^	AACC**C**CTA	SRp55	3.89	12
USMG5	rs7911488	D	C	CTG**C**CAA	SF2/ASF^6^	2.40	1186
				TGCTG**C**	SRp55	3.02	
			T^5^	None	-	-	282
ESPL1	rs6580942	B^7^	A^5^	TGCCCG**A**	SF2/ASF^6^	2.19	145
				CCG**A**CTT	SF2/ASF^6^	2.57	
				G**A**CTTGAA	SC35	2.71	
				CG**A**CTTG	SRp40	2.88	
			C	CCG**C**CTT	SF2/ASF^6^	2.16	418
MMAB	rs2287180	C	T	CTGCC**T**A	SF2/ASF^6^	2.64	217
				C**T**ACTCT	SRp40	2.72	
				TGCC**T**A	SRp55	3.47	
			C^5^	CTGCC**C**A	SF2/ASF^6^	2.53	81
				C**C**ACTCT	SRp40	3.03	
RBCK1	rs41281892	C	G^5^	CTGAG**G**T	SF2/ASF^6^	4.15	95
				CCTGAG**G**	SRp40	3.20	
			A	CTGAG**A**T	SF2/ASF^6^	2.37	5
CCDC41	New SNP at	C/D	G	G**G**ATCTTA	SC35	3.03	84
	chr12:93353207			TGAG**G**A	SRp35	2.82	
			C	AG**C**ATC	SRp55	3.63	32
ZNF83	rs7248435	F^7^	C	TGTGG**C**	SRp55	3.77	13
			A^5^	TGTGG**A**	SRp55	3.41	56
ATP5SL	rs1043413	D	C^5^	**C**CCACGT	SF2/ASF^6^	4.49	379
				T**C**CCACG	SRp40	3.62	
				TGCT**C**CCA	SC35	3.07	
			G	TGCT**G**C	SC35	3.29	153
				T**G**CCACG	SRp40	2.80	
RNASEN	rs55656741	D	C^5^	TTTAT**C**G	SRp40	2.84	307
			T	GTTTAT**T**G	SC35	2.80	174
				TTAT**T**GG	SRp40	3.35	
SERGEF	rs211146	C	A^5^	TTC**A**TC	SRp55	3.22	219
			G	C**G**TCTCCG	SC35	2.45	129
				C**G**TCTCC	SRp40	2.70	
				TTC**G**TC	SRp55	3.83	
ACP1	rs11553746	D	T	None	-	-	423
			C^5^	TGA**C**AGC	SRp40	4.66	269

Note: The relative splice-site usage disagrees with expectations for the last 3 cases.

1 See Figure 2. Cases marked C/D are cases where the SNP is very close to the middle of the exon.

2 Bold font indicates the SNP position.

3 Score calculated using ESE Finder 3.0 online tool [16].

4 Averaged PLIER-summarized expression score for each homozygous genotype.

5 Ancestral genotype, as inferred from the chimpanzee genome.

6 SF2/ASF (IgM-BRCA1) [51].

7 Cases for which an inverse correlation between splicing efficiency and probeset expression is expected based on the two isoform structures and the position of the affected splice-site, as shown in Figure 2.

Out of the 4 validated and sequenced AS regions remaining, 1 had a SNP inside the retained intron (HNRPH1) and 3 had one or more intronic SNPs around the cassette exon (IL6, WDR67 and SIDT1). It is likely that intronic SNPs play a role in determining isoform levels through their effect on intronic splicing enhancers (ISEs) or silencers (ISSs). We used another online software tool, SpliceAid [17], to detect ISEs or ISSs but only one of the 3 cases, showed qualitative agreement with the expression data (data not shown), indicating that we are either looking at the wrong candidate SNPs or that our understanding of ISEs/ISSs is not yet detailed enough to predict the effect of all these polymorphisms. Since we only sequenced approximately 200 base pairs from each exons, and introns generally span thousands of bases, causative SNPs are likely to be found further away in the intron. Intronic SNPs were found in many cases in conjunction with exonic SNPs, but there were often multiple intronic SNPs. Since we only looked for qualitative agreement between *in silico* predictions and the observed splicing differences, looking at many SNPs per gene would surely have caused more chance correlations and we would have been unable to rank the effects of SNPs affecting different splicing factors. For this reason, we prioritized exonic or splice-site bordering SNPs, for which there was almost always a single candidate.

### *In vivo* validation of causative SNPs

**Figure 3:**
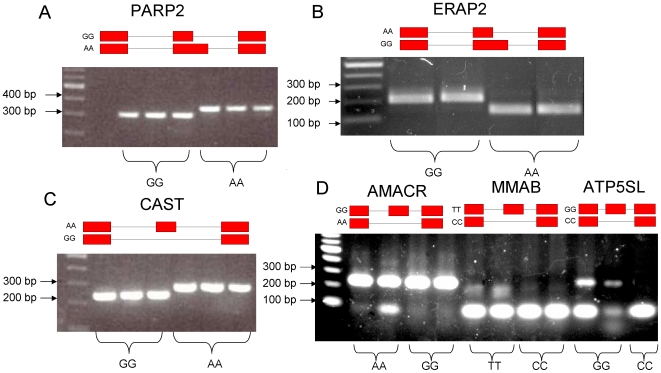
Electrophoresis bands of RT-PCR–amplified minigene mRNA products. Each column represents a different individual of the specified genotype. Above each gene's respective bands are the abstract depiction of the expected isoform structures associated with each genotype, boxes indicating exons and lines, introns (the relalive lengths are not to scale). For (A–C), the causative SNPs are, respectively, rs2297616, rs2248374 and rs7724759, which all affect the splice-site region, as described in Table 2. In these cases, the band migrations demonstrate that the individual genotypes are tightly linked with a complete change in isoform length. In these cases, only the associated isoform is expected to be present in individuals of a specific homozygous genotype. In (D), the causative SNPs for AMACR and ATP5SL are, respectively, rs10941112 and rs1043413, which are exonic and disrupt ESE sequences, as described in Table 3. While for MMAB, it can be one of 3 consecutive, fully linked SNPs, the prime candidate being rs2287180, which also disrupts ESE sequences (see Table 3). In these last 3 cases, instead of a complete switch in isoform length, we observe a change in the intensities of detectable isoforms which is perfectly associated with the SNP genotype. This makes sense considering the relatively less crucial role of ESEs as compared to the immediate splice-site neighborhood. The isoform structures depicted represent the isoform structure which is favored in each genotype, relative to the other genotype. All of the first columns on the left are 100 bp increment reference ladders.

In order to assess whether the candidate SNPs truly cause the splicing differences *in vivo*, for 6 genes we sub-cloned the exon and surrounding intronic sequence from individuals of different genotypes into a minigene expression vector [18]. Placing the exon of interest between two constitutive exons within the reporter construct allows determining the effect of the SNP on candidate exon inclusion levels. We used sequencing to verify that the sub-cloned construct differed only at the SNP positions we had previously predicted to be responsible for the differential splicing event. In 5 out of the 6 cases, the putative causative SNP was the only SNP present. MMAB however contains 3 closely neighboring SNPs which are perfectly linked. We transiently transfected the constructs into HeLa cells and assessed the presence of different isoforms using RT-PCR. Figure 3 shows the electrophoresis band migrations along with the SNP genotypes. For CAST, ERAP2, and PARP2, for which the candidate SNP is very close to the splice-site, we demonstrate that the predicted SNP causes a complete switch in 5′ splice-site usage (PARP2, ERAP2, Figure 3A and Figure 2B) or a complete skipping of the exon (CAST, Figure 3C). In the 3 other cases, ATP5SL, MMAB and AMACR, for which the candidate SNPs were found in the exon and were predicted to disrupt ESEs, the assay shows a visible but subtle change in isoform ratios (Figure 3D) in the expected direction. We used the Agilent 1000 DNA chip to quantify the results for these three cases because their gel bands are less convincing than the first three cases. We included the capillary electrophoresis readings as Figure S1. The quantification of the isoforms from the peaks show that the smallest difference between individuals of different genotypes, the first and last columns for the AMACR gene in Figure 3, consists of a 1.5 fold difference in isoform ratios, confirming that all the changes in isoform ratios were measurable and in the expected direction. Why significant variation exists between individuals with the same genotype is hard to say, especially considering that the plasmid inserts were confirmed to have the same sequence. The differences must come either from changes incurred during transfection (a single transfection assay was performed for each plasmid) or from biological or technical noise. Except for MMAB, for which any one of the 3 SNPs or even a combination of all 3 could be causative, these results demonstrate the causative nature of our candidate SNPs and confirm that both systematic splicing differences as well as more subtle, quantitative, differences exist between individuals and the extent of the change is reflective of the position of the causative SNP and of *in silico* predictions of its effect.

### Detecting signs of positive selection

In order to gain insight into the persistence within human populations of SNPs with such drastic effects on the structure of the expressed transcripts, we wanted to assess whether some of the causative SNPs showed signs of recent positive selection. We performed two tests. Using the program fdist2 [19], we calculated the fixation index (Fst) of all 41 re-sequenced HapMap SNPs based on the frequencies in the 4 available populations (see Methods), and compared the results to a simulated dataset. The average Fst was significantly greater than expected (p = 0.046) by a small margin. One SNP had a Fst above the 99.999^th^ percentile relative to the simulation dataset, rs6580942 (A/C), the best candidate SNP in the ESPL1 gene. This is very significant, even in a sample of 41 (p = 4.1E-3). The C allele has a frequency of 0% in the African (YRI) and Asian (CHB+JPT) populations compared to 33% in the Caucasian population (CEU). We then calculated the highest relative extended haplotype homozygosity (REHH) in the CEU population considering various size blocks below 500 kb centered on the same 41 SNPs (see Methods for details). We performed the same analysis on 300 randomly chosen SNPs in genes as a control. There was no significant difference between the average highest REHH of our sequenced SNPs and the control set. No SNP showed a REHH score above the 99^th^ percentile of the control set. However, we noticed that rs10941112 (A/G), the best candidate SNP from the AMACR gene, had both a Fst above the 97^th^ percentile, with the A genotype varying from 0% in the African population to 37% and 62% in the Asian and Caucasian populations respectively, and a REHH above the 97% percentile, with the A allele showing 122.5 fold greater homozygosity for a block of 391 kB around the SNP than the G allele. The combined extreme result in both tests is highly unlikely (p = 8.0E-4) and is indicative of positive selection. These two analyses have brought to light strong evidence suggesting that the SNPs rs6580942 and rs10941112, in the ESPL1 and AMACR genes, respectively, have undergone recent positive selection. Other than these 2 SNPs, there is no strong evidence of positive selection, indicating that many of our re-sequenced HapMap SNPs may be selectively neutral.

## Discussion

### Can we tell how much splicing variation exists between individuals?

Previous micro-array studies have often attempted to estimate the real amount of AS or gene-level differences simply from counting the number of cases which surpass a multiple-testing corrected significance threshold [6],[8], placing complete faith in the results of the micro-array as well as the normalization, summarization and whichever AS detection algorithm was used. The first problem with such an approach is that many sources of noise can cause false positives, like SNPs within probes, cross-hybridizations or technical noise, as well as other distortions such as probesets responding unevenly to a gene-level change. The second weakness of such estimations, as our results demonstrate, is that many real AS events lie far below standard significance thresholds. The highest P-value for a validated AS candidate in this study was 7.3E-4 as opposed to 4.2E-9 in the previous study on the same dataset. This means that approximately 15 times more probesets could be considered potential AS candidates. Of course, we do not suggest that we found 15 times more AS than the previous study but rather, by integrating EST evidence and utilizing a more sophisticated AS detection algorithm, we can show that the speculative content of the array, which comprises about 80% of the array, as well as the less significant measurable differences on the array contain valuable information which have thus far, been mostly overlooked. Our results show that Exon-Array data by itself may be too noisy to produce reliable estimates of AS at the genome-wide scale. All we can conclude is that genetically controlled splicing differences exist between individuals and are probably more common than was previously estimated. A more definitive answer on the real extent of individual-specific AS may come from high-throughput sequencing, which can avoid probe target bias and detect AS differences qualitatively rather than through statistical inference.

### Alternative splicing and disease predisposition

We have shown that many splicing differences between healthy individuals can be identified using the Exon Array platform. We expect that many more, perhaps approaching the complete map of the splicing eQTL (expression quantitative trait loci) landscape, will be catalogued soon using more sensitive methods, such as deep mRNA sequencing. Most of these splicing differences which we can detect are controlled by polymorphisms in cis-regulatory regions or in the vicinity of an implicated splice-site. These differences between individuals could contribute to phenotypic variation and could either be neutral in their effects, or confer differential susceptibility to complex diseases. A few of our validated events occur in genes which have already been associated with diseases. BCKDHA is related to maple syrup urine disease, type 1a [20], a rare inherited metabolic disorder which, without a highly controlled diet and close monitoring of blood chemistry, causes progressive neurological damage which can cause vomiting, eating difficulties, irregular breathing, coma or death. Deficiency of the gene alpha-methylacyl-coa racemase (AMACR) is a rare disorder of the fatty acid metabolism which is characterized by neuronal and liver abnormalities [21] and the gene is considered a useful biomarker for various types of cancer [22], making it quite interesting that this gene contained the SNP with the most evidence of positive selection. Interleukin 6 (IL6) is an important mediator of fever [23] and the gene has been associated with osteoporosis [24] and Kaposi's sarcoma [25]. MMAB is related to vitamin B12 responsive methylmalonic aciduria [26], the inability to synthesize adenosylcobalamin, a vitamin B12 derivative, and whose symptoms include metabolic acidosis and retarded development. A SNP in MMAB, which is in linkage disequilibrium with our causative splicing SNP, has recently been associated with HDL cholesterol levels [27]. Surprisingly, all of the above AS events are within protein-coding regions of the genes, making it very likely that these heritable differences contribute to individuals' predisposition to disorders similar to those caused by inactivation of those genes or to other, more complex, diseases.

Complex diseases such as diabetes, cancer or schizophrenia are expected to be influenced by polymorphisms in a large number of genes, which may interact in multifarious ways. Many of the polymorphisms we identified in this study induce, through AS, potentially much more dramatic changes to the protein sequence than do non-synonymous coding SNPs. We have shown that common SNPs can influence alternative splicing across individuals even in relatively important genes, indicating that the genotyping of such SNPs will likely play a very significant role in predicting the occurrence of complex diseases in the future.

### Isoform eQTLs and the emerging role of *in silico* predictions

Hundreds of genome-wide association studies (GWAS) carried out to date have generally failed to identify causative protein-coding disease variants [28]. Many of the underlying causes may be due to subtler, regulatory genetic influences [29]. Thus, a lot of interest and resources have been allocated to identifying eQTLs, genes whose expression levels are affected by regulatory SNPs. Although identifying eQTLs has been quite successful [6], [30]–[33], there has been considerably less accomplishments in teasing apart regulatory haplotypes and pinpointing the SNP actually responsible for the regulatory difference. Our group's earlier work demonstrated the existence of common isoform eQTLs; i.e. genes under genetic control resulting in differential expression of transcript isoforms including: alternative splicing, alternative polyadenylation, and alternative transcript initiation. Here, we postulate that in many of those cases, it should be possible to narrow down the regulatory region to the vicinity of the alternative event (e.g. cassette exon), and subsequently identify the causative polymorphism with high confidence. In some of the cases, as in the ERAP2 gene, the common splicing polymorphism introduces a premature stop codon, most likely resulting in nonsense-mediated decay of the alternative product and a drastic reduction in the overall transcript levels, suggesting that splicing and RNA processing variations may be underlying some of the common expression QTLs. This knowledge will be essential for future functional studies and perhaps for future applications such as genetic therapy.

In 2004 Nembaware et al. used public EST data to show the existence of allele-specific transcript isoforms in human [34]. In 2007, Hull et al. showed that it is possible to identify such events in lymphoblastoid cell lines [7]. They selected 70 alternative splicing events, and showed that 6 of them were consistently associated with a specific genotype. They also used in vivo assays demonstrating causative nature of 2 candidate SNPs, suggesting that a substantial number of alternative splicing events may be controlled by genetic polymorphisms. These studies were followed by genome-wide microarray-based approaches [6],[8],[35], which confirmed and further expanded our knowledge of genetic control of isoform variation. Earlier this year, Zhang et al. published an article [8] where they used the Exon-Array on lymphoblast cell lines to look for genetic variants which account for AS differences between populations. They claim, based on multiple-testing-corrected statistics, that they discovered 397 such differences between the Caucasian (CEPH) and African (YRI) populations. Recently, other groups have approached the problem from the purely genetics angle: knowing the polymorphisms that are present, they attempted to predict their effect computationally and identify exons that are differentially spliced across individuals. This approach has so far met with limited success. Elsharawy at al. [36] have obtained an extremely low validation rate of their *in silico* predictions, ranging from 0% for ESE predictions to 9% for SNPs in splice-sites, demonstrating our far from complete understanding of the effects of cis-regulatory sequences on splicing.

Thus, in the present study, we take further steps towards optimal integrated use of the existing data – gene structure annotation, splicing-sensitive microarray data, SNP databases and targeted sequencing - to detect splicing eQTLs and their genetic determinants. First, taking advantage of the high level of coverage of the current sequence-based annotation of AS events, we concentrate only on events that have been previously reported. This approach is highly justified by the observation from the previous analysis [6] that less than 10% of the detected and validated AS events were novel (unannotated), suggesting that the current EST coverage of the transcriptome is nearly complete. Secondly, we use a much improved algorithm to detect AS events in exon array data. Finally, we show that among the events that are detected using the above criteria and further validated in the lab, a majority contain SNPs that have highly suggestive *in silico* evidence of causation. We can show in a *post hoc* analysis that knowing the sequence variation information before-hand could have significantly improved the specificity of our search for AS. 27 out of 34 (79%) validated and sequenced alternatively spliced regions contained a SNP for which *in silico* evidence appropriately explained the change, compared to 2 out of 7 (29%) for the sequenced regions which were negatively validated (data not shown). Given the current low resolution of HapMap SNPs, making use of *in silico* predictions of SNPs' effects at the genome-wide level would only be applicable to a fraction of Exon Array probesets. Less than half of our likely causative SNPs (in splice-sites or exons) were HapMap SNPs. The experience of Elsharawy et al. showed that the specificity of the purely computational approach is quite low, given the current level of understanding of AS regulation. However, once the resolution of SNPs and their genotypes increases significantly, which is already the case for four CEPH HapMap individuals which were recently fully sequenced (http://www.1000genomes.org/), it should become feasible to merge computational predictions with biological expression data to improve the power to detect cis-acting polymorphisms involved in splicing. In turn, the identified polymorphisms and their effects will help to further enhance our understanding of splice-sites and cis-regulatory sequences.

### Understanding the “splicing code”

Out of the two splice-sites, the 5′ intronic splice-site (the donor site) appears to be the dominant identifiable target of these regulatory polymorphisms, with 14 predicted causative SNPs, as opposed to 3 for the 3′ splice-site (the acceptor site). Given that we considered 23 bases for the 3′ splice-site compared to 9, for the 5′ splice-site, it seems unlikely the result of pure chance, suggesting either that there may be greater purifying selective pressure acting on the region around the 3′ splice-site, or that, due to the greater degeneracy of the 3′ sequence [15], SNPs affecting it influence splicing strength too subtly to be detected by the micro-array platform. In the case of cassette exons, the fact that the 5′ splice-site sequence, as well as ESE sequences, influence the use of the upstream splice-site defining the exon start, demonstrates the fact that in multi-intronic genes in vertabrates, the “exon definition” step, whereby splice-sites are paired across the exon, takes place before the assembly of the mature spliceosome and splicing of the intron can occur [37],[38], as opposed to species like yeast, whereby the intron definition occurs independently for each intron and a mutation of the 5′ splice-site would cause retention of the downstream intron rather than exon skipping [39]. The fact that the vast majority of putative causative SNPs affected either the 5′ splice-site or ESE sequences shows that the exon definition plays a central role in generating these splicing variations across individuals.

### Conclusion

Expression QTL analysis has garnered considerable interest in recent years and is increasingly being used in conjunction with whole genome association studies to narrow down the list of genetic variants putatively responsible for complex genetic disorders [28]. Here, we focus on a specific type of eQTL, alternative splicing variation, and extend the results of prior studies by validating the greatest number of these differences and showing that such variation may be more common than previously estimated, and that its effects can be quantitatively very subtle. Furthermore, this work demonstrates that we have the ability to identify the specific causative genetic variants responsible for isoform eQTL differences among individuals. It also underscores the value of data integration in order to obtain improved true positive rates in large scale analyses of splicing. With the upcoming release of the 1000 genomes data [40] and the growing use of high-throughput sequencing for transcriptome analysis, we will be able to broaden our understanding of the complex intricacies of the “splicing code” and use this knowledge to confidently identify splicing regulatory SNPs.

## Methods

### Integrating public AS information and mapping to exon-array probesets

In order to select alternative splicing candidates within the vast set of significant associations between SNPs and probeset expression, we used publicly available knowledge of AS events to prioritize the list. We only considered Exon-Array probesets whose target coordinates overlapped an EST-supported AS event. We used a cut-off of a single base, reasoning that a single base mismatch could significantly affect the probe binding efficiency [41]. We downloaded the list of putative AS events in Human based on UCSC Known genes and human AS events from ASAP-II. We also retrieved from the UCSC Genome Browser website the full tables describing the human genome annotations based on Blated [42] spliced EST and mRNA sequences to gather some additional AS evidence.

We applied strict criteria when inferring potential AS events from EST/mRNA data. To avoid confusing intron retention events with incomplete splicing or transcript length changes with incomplete mRNA sequences, we only selected cassette exons or alternative splice site usages. The latter two types of AS are also most confidently validated via PCR since they generally introduce short changes in the mRNA sequence. We had to define a reasonable gene structure from the Blat results, which, especially for EST annotations, include many short gaps which most probably originated from sequencing errors rather than genuine splicing. We considered gaps greater than 30 bps as introns, ignored gaps smaller or equal to 3 bases, and filtered out annotations containing gaps of any size in between. An exon was defined as a continuous alignment of 15 bases or longer, surrounded in the contig by an intron and a part of an exon on each side. Alternative splice-site usages had to be at least 6 bases long and their length a multiple of three if they fell within a UCSC gene's coding region. We insisted that EST's or mRNA's supporting an event should contain sequence from the next exon on each side of the event with the flanking introns spliced. Since we considered EST data to be less reliable than the mRNA data, we only reported events with at least two EST's supporting each isoform.

### Identifying genetically-controlled isoform variations

This step was divided into two parts: the analysis of the core probesets, the roughly 240,000 probesets that target well supported exons, and the analysis of all probesets, which includes many more potential genes and gene regions with little evidence of ever being expressed. In both cases the algorithm was the same but we modified the thresholds in order to take into account the greater amount of noise from the non-core probesets. The normalization, summarization and regression steps were described in an earlier publication [6]. Briefly, PLIER was used to normalize the data and summarize the probe scores into probeset scores and linear regression was used to correlate the expression level of each probeset with the genotypes of every known SNP within 50kB of the gene.

Subsequently, for each gene (meta-probeset), we identified the most significant probeset-SNP association and used this SNP to report a regression p-value and fold-change between homozygous genotypes for every probeset in the gene. For SNPs with no homozygous minor allele individuals, the expression fold-change between the two genotypes was doubled in order to estimate the predicted fold-change between homozygotes. To choose probesets as candidates for AS, we applied a threshold p-value for the linear regression and an absolute value threshold for a score U, as described in Equation 1, which represents the unexpectedness of the fold-change in the context of the other probeset expression changes in the gene.
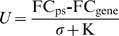
(1),where, in log_2_ values, FC_ps_ is the probeset fold-change, FC_gene_ is the average fold-change of all other probesets in the gene, σ is the standard deviation of the other fold-changes and K is a small constant which we arbitrarily set at 0.2, added to control for genes with too little variation, or in other words, stabilize the variance.

For probesets that passed both thresholds and had prior AS evidence, we plotted the log_10_ of the p-values and the log_2_ of the fold-changes of all probesets in the gene onto the UCSC Genome Browser as custom tracks and selected candidates for PCR validation via visual inspection. Links to all selected candidate tracks are available in Table S1. The “AS-marker” track indicates the position of the affected probeset. We visually inspected 100 candidates from the analysis of core probesets and 160 candidates from the analysis of all probesets, in order to select only the ones where the microarray based and EST-based predictions where concordant in the context of the entire transcript. For the visually verified candidate probesets, we re-applied the linear regression using the probe-level scores and retained only the candidates for which a majority of individual probes agreed with the probeset-level regression (which is based on PLIER's probeset summary scores).

The unexpectedness (U) measure described in Equation 1 is better suited at finding short AS events, such as single cassette exons or alternative splice site usage rather than large splicing changes, such as the one observed in the ELAVL1 gene in the previous paper [43], where half of the probesets can be included or excluded. This later case would cause the standard deviation of fold-changes to be very large, thus bringing down the score. We were specifically looking for short AS events since they are more readily amplifiable via PCR and hence easy to validate. The presence of the standard deviation in the denominator also helps to avoid noisy genes due to low expression or inconsistent splicing patterns, such as immunoglobulin genes, and false positives caused by the erroneous inclusion of multiple genes in the same meta-probeset.

### PCR validation

We selected 72 potential AS events for PCR validation. Although we had all 57 lymphoblast samples in our lab's possession, given limited resources, we selected a strategic sample of 10 individuals which together harbored variation for all 72 associated SNPs and forwarded the samples for validation of the alternative splicing events using semi-quantitative PCR. Primers were successfully designed for 62 events, but 4 of the reactions did not yield any product (primer failure). The sizes and relative quantity of amplicons were determined using a Caliper LC90 automated chip-based capillary electrophoresis instrument and then sizes were matched-up with expectations based on AceView gene annotations, as detailed in the paper describing this automated AS validation procedure [44]. For the 58 reactions which produced viable results, we performed linear regression on the estimated ratios of each amplicon with the genotypes of the associated SNP. When the direction of the slopes and the lengths of isoforms agreed with the micro-array regression and the smallest regression p-value was below 0.05, we considered these cases fully validated as genetically cis-regulated isoform level variations. If the lowest p-value was above 0.05 but there was a clear visible trend in the right direction, we classified them in the second category: not statistically significant, but two isoforms were present with a trend in the right direction. We also designated lower levels of validation confidence for cases that showed isoform level variations but where the association with the SNP could not be verified, and finally for cases that showed AS without detectable inter-individual variation.

### Sequencing

We performed sequencing of the genomic DNA of the regions around the 72 validation candidate AS events as well as 10 candidate events from previous analyses. We selected 4 individuals to sequence for every AS candidate exon. We chose 2 individuals to represent each distinct homozygous genotype and made sure they also represented the difference in probeset expression associated with the genotype. Heterozygotes were used in cases where the rare minor genotype was not present in our sample. We designed primers with Primer3 [45] to amplify a region of 600–800 bases centered on the probeset and sequenced these regions in the 4 individuals. In total, we retrieved useful sequence information for 60 of the regions and we were able to detect 86 SNPs. We used Phred [46], Polyphred [47] and Consed [48] to analyze the chromatograms and we aligned the 4 output sequences to a reference sequence using ClustalW [49] to map the detected SNPs to the genome. To view the SNPs in the context of the gene structure and identify potentially important polymorphisms, we displayed the SNP positions with their associated micro-array expression change on the UCSC Genome Browser as custom tracks. These tracks are available in the Table S2.

### Scoring polymorphic splice-sites and splice-site motifs

Whenever a SNP was close enough to a splice-site, we used the MaxEntScan [15] online tool to score the theoretical strength of the different versions of the splice-site sequence. We found 13 SNPs that fell inside MaxEntScan's 9 base window around the 5′ splice-site and 3 SNPs that fell inside the 23 base window around the 3′ splice-site (see Table 2). In every case, we could show a qualitative agreement between the expected change in splice-site strength calculated by MaxEntScan and the micro-array expression change.

### Predicting and scoring SNP-affected exonic splicing enhancers

For every SNP that was sequenced in an exon, we used the online tool ESE Finder 3.0 to predict potential exonic splicing enhancers (ESEs) that would be affected by the SNP (see Table 3). We tabulated the sequences and matrix-based scores of all ESE predictions containing the polymorphic base and surpassing ESE finder's established thresholds, except for the one ESE detected in the WARS gene, which was only 0.04 below the default threshold.

### Minigene assays

We made use of *in vivo* minigene assays to verify the causal link between 6 candidate SNPs and detected AS events. The minigene assays were based on an article by Singh and Cooper [18]. We performed the experiment on 2 genes from this analysis, MMAB, and AMACR, and 4 top-scoring hits from the previous study, PARP2, ERAP2, ATP5SL and CAST. Primers were designed to amplify the alternatively spliced exon and 200bps from flanking introns. Genomic DNA was amplified for four individuals in each case, two with each homozygous genotype for the SNP of interest. Sequences were digested with Xba1 and Sal1, as was the RHCGlo plasmid vector. The plasmid and amplicons were then ligated. Clones were purified, PCR-amplified and sequenced, to verify that the final plasmid sequences assayed differed only at the expected SNP. Then 2ug of the new plasmids were transfected into wells containing ∼3×10^15^ HeLa cells. 24–48h after transfection, RNA was extracted using Trizol, following the manufacturer's instructions. Alternative splicing was assessed via RT-PCR using the plasmid-specific primers RSV5U and TNIE4 [18]. Isoform presence was assessed by gel electrophoresis (see Figure 3) and, in 3 cases (AMACR, MMAB, ATP5SL) quantified by capillary electrophoresis using the Agilent DNA 1000 Chip Kit, according to the manufacturer's protocol.

### Fixation index (Fst)

We used the publically available program fdist2 [19] to calculate the Fst of all 41 HapMap SNP we sequenced based on the genotype frequencies in 4 populations, CEPH, YRI, CHB and JPT, merging the Asian population (CHB and JPT) frequencies together, as recommended by the fdist2 instructions. In order to estimate the percentile ranking of our SNP Fsts, we used fdist2 to create a simulation dataset of 20,000 data points based on 3 demes, 3 sample populations and an expected Fst of 0.187, which was the average Fst estimated from 800 random HapMap SNPs.

### Relative extended haplotype homozygosity (REHH)

For all 41 re-sequenced HapMap SNP, based on the phased haplotypes of the CEU HapMap population (Build 36) and a core haplotype of a single SNP, we measured the relative extended haplotype homozygosity, as defined by Sabeti et al. [49], at every size below 500 kb, each step extending the haplotype by one SNP on each side. We reported the highest measured REHH for any block size, which was generally situated between 100 and 400 Kb. The minor core haplotype frequency had to be above 0.2 because lower frequencies can cause artifactually high REHH values [50]. We also insisted that REHH measurements be supported by at least 10 identical phased haplotypes to avoid performing the measurements on overly fragmented haplotypes. We performed the same analysis on 300 randomly chosen HapMap SNPs that fell within a RefSeq gene and used this distribution to estimate the relative percentile of the maximum REHHs of our sequenced SNPs.

## Supporting Information

Figure S1 Capillary electrophoresis readings from the Agilent 1000 DNA chip for the minigene assays. FU stands for fluorescence units. Each column shows the readings for all assays for a specific gene. The title of each graph denotes the individual from which the plasmid insert was derived and his genotype for the SNP of interest. The individuals, from top to bottom, are in the same order as the gel columns in Figure 3, from left to right. The arrows point to the peaks assigned to the two isoforms. The first and last peak are lower and upper markers, respectively.
(1.01 MB TIF)

Table S1 Events selected for RT-PCR validation.
(0.15 MB DOC)

Table S2 SNPs discovered around AS events.
(0.09 MB DOC)
