# Supplementary figures and images for: Fine-Scale Variation and Genetic Determinants of Alternative Splicing across Individuals
Source: PLoS Genet. 2009 Dec 11;5(12):e1000766. doi: 10.1371/journal.pgen.1000766 (PMC2780703; doi:10.1371/journal.pgen.1000766)

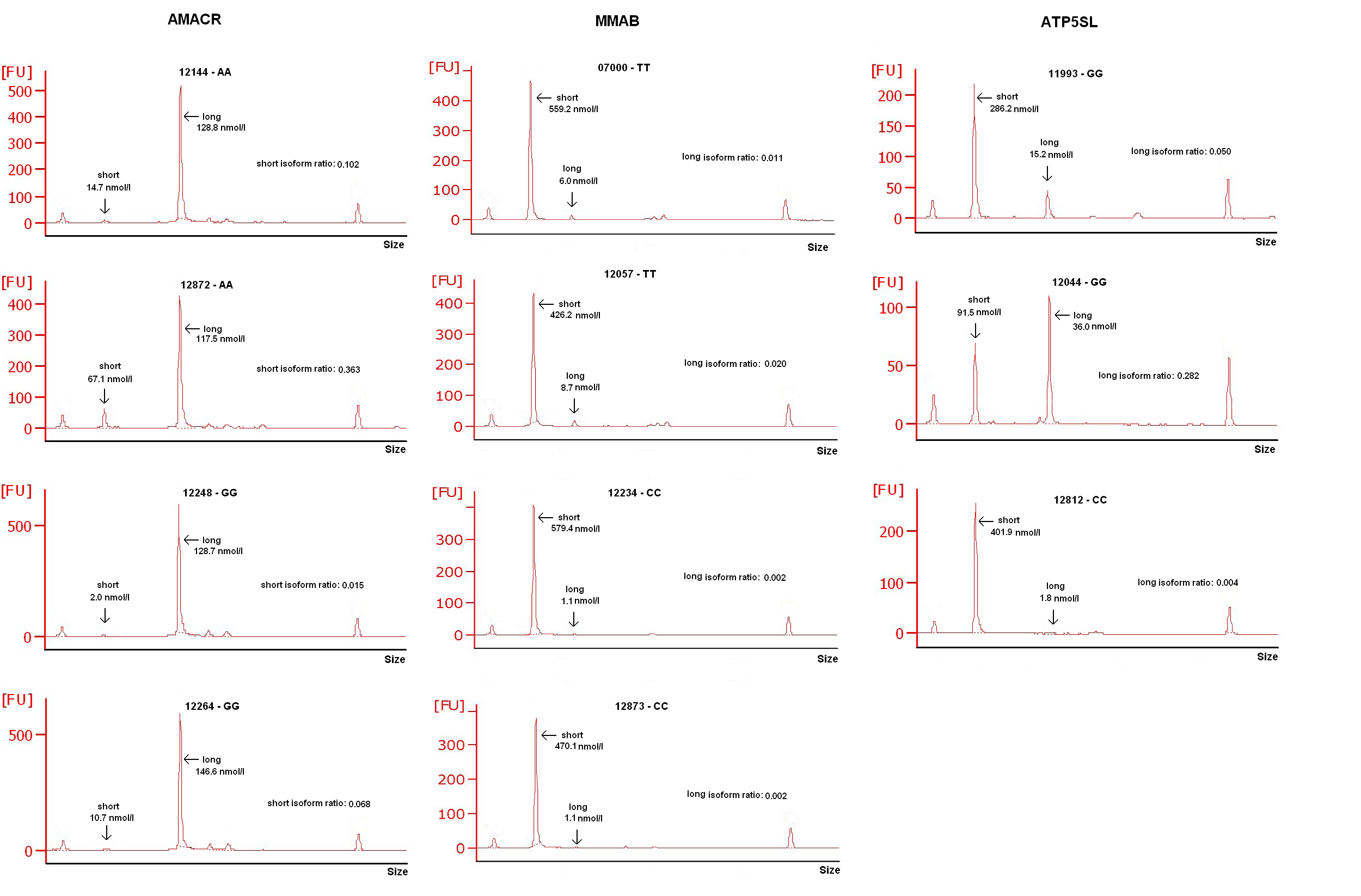

Supplement: Figure S1 — Capillary electrophoresis readings from the Agilent 1000 DNA chip for the minigene assays. FU stands for fluorescence units. Each column shows the readings for all assays for a specific gene. The title of each graph denotes the individual from which the plasmid insert was derived and his genotype for the SNP of interest. The individuals, from top to bottom, are in the same order as the gel columns in Figure 3, from left to right. The arrows point to the peaks assigned to the two isoforms. The first and last peak are lower and upper markers, respectively. (1.01 MB TIF) [file pgen.1000766.s001.tif]
