# Supplementary material for: Fine-Scale Variation and Genetic Determinants of Alternative Splicing across Individuals
Source: PLoS Genet. 2009 Dec 11;5(12):e1000766. doi: 10.1371/journal.pgen.1000766 (PMC2780703; doi:10.1371/journal.pgen.1000766)
Supplement: Table S1 — Events selected for RT-PCR validation. (0.15 MB DOC) [file pgen.1000766.s002.doc]

Table S1: Events selected for RT-PCR validation

| Gene | Probeset | Analysis | SNP | Browser |
| --- | --- | --- | --- | --- |
| ZNF695 | 2465409 | all | rs2818869 | [2465409](http://genome.ucsc.edu/cgi-bin/hgTracks?org=human&db=hg17&position=chr1:243434907-243700162&hgt.customText=http://genomequebec.mcgill.ca/majewski/jas_UCSC/reg/2465409) |
| NT5DC3 | 3468757 | all | rs12368209 | [3468757](http://genome.ucsc.edu/cgi-bin/hgTracks?org=human&db=hg17&position=chr12:102667085-102737459&hgt.customText=http://genomequebec.mcgill.ca/majewski/jas_UCSC/reg/3468757) |
| ESPL1 | 3415861 | core | rs2016266 | [3415861](http://genome.ucsc.edu/cgi-bin/hgTracks?org=human&db=hg17&position=chr12:51948395-51973593&hgt.customText=http://genomequebec.mcgill.ca/majewski/jas_UCSC/as/3415861) |
| RGL3 | 3850922 | all | rs4804592 | [3850922](http://genome.ucsc.edu/cgi-bin/hgTracks?org=human&db=hg17&position=chr19:11348918-11390990&hgt.customText=http://genomequebec.mcgill.ca/majewski/jas_UCSC/reg/3850922) |
| SH3YL1 | 2537134 | core | rs9213 | [2537134](http://genome.ucsc.edu/cgi-bin/hgTracks?org=human&db=hg17&position=chr2:208176-254022&hgt.customText=http://genomequebec.mcgill.ca/majewski/jas_UCSC/as/2537134) |
| C8orf59 | 3142947 | all | rs3808538 | [3142947](http://genome.ucsc.edu/cgi-bin/hgTracks?org=human&db=hg17&position=chr8:86313463-86319831&hgt.customText=http://genomequebec.mcgill.ca/majewski/jas_UCSC/reg/3142947) |
| XPNPEP3 | 3946515 | all | rs4821976 | [3946515](http://genome.ucsc.edu/cgi-bin/hgTracks?org=human&db=hg17&position=chr22:39577603-39653299&hgt.customText=http://genomequebec.mcgill.ca/majewski/jas_UCSC/reg/3946515) |
| IFI44L | 2343481 | core | rs273238 | [2343481](http://genome.ucsc.edu/cgi-bin/hgTracks?org=human&db=hg17&position=chr1:78798194-78819686&hgt.customText=http://genomequebec.mcgill.ca/majewski/jas_UCSC/as/2343481) |
| HNRPH1 | 2890160 | all | rs7442819 | [2890160](http://genome.ucsc.edu/cgi-bin/hgTracks?org=human&db=hg17&position=chr5:178970170-178984260&hgt.customText=http://genomequebec.mcgill.ca/majewski/jas_UCSC/reg/2890160) |
| TCP11 | 2951389 | all | rs4711408 | [2951389](http://genome.ucsc.edu/cgi-bin/hgTracks?org=human&db=hg17&position=chr6:35193872-35288530&hgt.customText=http://genomequebec.mcgill.ca/majewski/jas_UCSC/reg/2951389) |
| SGOL1 | 2665585 | all | rs17006682 | [2665585](http://genome.ucsc.edu/cgi-bin/hgTracks?org=human&db=hg17&position=chr3:20177209-20215520&hgt.customText=http://genomequebec.mcgill.ca/majewski/jas_UCSC/reg/2665585) |
| SLC3A2 | 3333716 | core | rs3763851 | [3333716](http://genome.ucsc.edu/cgi-bin/hgTracks?org=human&db=hg17&position=chr11:62380154-62412771&hgt.customText=http://genomequebec.mcgill.ca/majewski/jas_UCSC/as/3333716) |
| GTF3C2 | 2545738 | core | rs11684134 | [2545738](http://genome.ucsc.edu/cgi-bin/hgTracks?org=human&db=hg17&position=chr2:27460380-27484903&hgt.customText=http://genomequebec.mcgill.ca/majewski/jas_UCSC/as/2545738) |
| FAM64A | 3707965 | all | rs7218283 | [3707965](http://genome.ucsc.edu/cgi-bin/hgTracks?org=human&db=hg17&position=chr17:6289120-6295477&hgt.customText=http://genomequebec.mcgill.ca/majewski/jas_UCSC/reg/3707965) |
| PPIL2 | 3938300 | core | rs5999098 | [3938300](http://genome.ucsc.edu/cgi-bin/hgTracks?org=human&db=hg17&position=chr22:20344850-20377060&hgt.customText=http://genomequebec.mcgill.ca/majewski/jas_UCSC/as/3938300) |
| P11 | 3452674 | core | rs12368459 | [3452674](http://genome.ucsc.edu/cgi-bin/hgTracks?org=human&db=hg17&position=chr12:46390075-46405617&hgt.customText=http://genomequebec.mcgill.ca/majewski/jas_UCSC/as/3452674) |
| SMAD5 | 2830027 | core | rs13160439 | [2830027](http://genome.ucsc.edu/cgi-bin/hgTracks?org=human&db=hg17&position=chr5:135496469-135546128&hgt.customText=http://genomequebec.mcgill.ca/majewski/jas_UCSC/as/2830027) |
| NHEDC1 | 2780084 | core | rs227375 | [2780084](http://genome.ucsc.edu/cgi-bin/hgTracks?org=human&db=hg17&position=chr4:104163886-104270430&hgt.customText=http://genomequebec.mcgill.ca/majewski/jas_UCSC/as/2780084) |
| DMKN | 3859789 | core | rs7245699 | [3859789](http://genome.ucsc.edu/cgi-bin/hgTracks?org=human&db=hg17&position=chr19:40680034-40696339&hgt.customText=http://genomequebec.mcgill.ca/majewski/jas_UCSC/as/3859789) |
| FAM107B | 3278834 | all | rs17155523 | [3278834](http://genome.ucsc.edu/cgi-bin/hgTracks?org=human&db=hg17&position=chr10:14589897-14856852&hgt.customText=http://genomequebec.mcgill.ca/majewski/jas_UCSC/reg/3278834) |
| ACP1 | 2466156 | core | rs9213 | [2466156](http://genome.ucsc.edu/cgi-bin/hgTracks?org=human&db=hg17&position=chr2:261866-268240&hgt.customText=http://genomequebec.mcgill.ca/majewski/jas_UCSC/as/2466156) |
| C14orf129 | 3550335 | all | rs2053588 | [3550335](http://genome.ucsc.edu/cgi-bin/hgTracks?org=human&db=hg17&position=chr14:95899178-95923376&hgt.customText=http://genomequebec.mcgill.ca/majewski/jas_UCSC/reg/3550335) |
| CDK7 | 2813489 | core | rs12656918 | [2813489](http://genome.ucsc.edu/cgi-bin/hgTracks?org=human&db=hg17&position=chr5:68566491-68608978&hgt.customText=http://genomequebec.mcgill.ca/majewski/jas_UCSC/as/2813489) |
| ZNF419 | 3843285 | core | rs6510083 | [3843285](http://genome.ucsc.edu/cgi-bin/hgTracks?org=human&db=hg17&position=chr19:62691131-62697724&hgt.customText=http://genomequebec.mcgill.ca/majewski/jas_UCSC/as/3843285) |
| VISA | 3874507 | all | rs8116776 | [3874507](http://genome.ucsc.edu/cgi-bin/hgTracks?org=human&db=hg17&position=chr20:3775476-3804745&hgt.customText=http://genomequebec.mcgill.ca/majewski/jas_UCSC/reg/3874507) |
| AZIN1 | 3147621 | all | rs2679757 | [3147621](http://genome.ucsc.edu/cgi-bin/hgTracks?org=human&db=hg17&position=chr8:103907725-103953916&hgt.customText=http://genomequebec.mcgill.ca/majewski/jas_UCSC/reg/3147621) |
| VEZT | 3426838 | all | rs3751271 | [3426838](http://genome.ucsc.edu/cgi-bin/hgTracks?org=human&db=hg17&position=chr12:94114020-94204711&hgt.customText=http://genomequebec.mcgill.ca/majewski/jas_UCSC/reg/3426838) |
| DUSP18 | 3957502 | all | rs5753268 | [3957502](http://genome.ucsc.edu/cgi-bin/hgTracks?org=human&db=hg17&position=chr22:29372737-29388402&hgt.customText=http://genomequebec.mcgill.ca/majewski/jas_UCSC/reg/3957502) |
| RNASEN | 2852054 | core | rs12513784 | [2852054](http://genome.ucsc.edu/cgi-bin/hgTracks?org=human&db=hg17&position=chr5:31436942-31567924&hgt.customText=http://genomequebec.mcgill.ca/majewski/jas_UCSC/as/2852054) |
| AMACR | 2852757 | core | rs3195676 | [2852757](http://genome.ucsc.edu/cgi-bin/hgTracks?org=human&db=hg17&position=chr5:34023057-34043934&hgt.customText=http://genomequebec.mcgill.ca/majewski/jas_UCSC/as/2852757) |
| PPIE | 2331628 | all | rs7520588 | [2331628](http://genome.ucsc.edu/cgi-bin/hgTracks?org=human&db=hg17&position=chr1:39827031-39898652&hgt.customText=http://genomequebec.mcgill.ca/majewski/jas_UCSC/reg/2331628) |
| MMAB | 3470844 | all | rs12580090 | [3470844](http://genome.ucsc.edu/cgi-bin/hgTracks?org=human&db=hg17&position=chr12:108456503-108474041&hgt.customText=http://genomequebec.mcgill.ca/majewski/jas_UCSC/reg/3470844) |
| MTMR12 | 2852298 | all | rs4562053 | [2852298](http://genome.ucsc.edu/cgi-bin/hgTracks?org=human&db=hg17&position=chr5:32262889-32348888&hgt.customText=http://genomequebec.mcgill.ca/majewski/jas_UCSC/reg/2852298) |
| RBCK1 | 3873192 | all | rs6051942 | [3873192](http://genome.ucsc.edu/cgi-bin/hgTracks?org=human&db=hg17&position=chr20:337184-359595&hgt.customText=http://genomequebec.mcgill.ca/majewski/jas_UCSC/reg/3873192) |
| SNORD49B | 3712109 | core | rs11869614 | [3712109](http://genome.ucsc.edu/cgi-bin/hgTracks?org=human&db=hg17&position=chr17:16283101-16285912&hgt.customText=http://genomequebec.mcgill.ca/majewski/jas_UCSC/as/3712109) |
| IKIP | 3467329 | all | rs2289315 | [3467329](http://genome.ucsc.edu/cgi-bin/hgTracks?org=human&db=hg17&position=chr12:97509757-97542264&hgt.customText=http://genomequebec.mcgill.ca/majewski/jas_UCSC/reg/3467329) |
| APPBP1 | 3695303 | all | rs16957014 | [3695303](http://genome.ucsc.edu/cgi-bin/hgTracks?org=human&db=hg17&position=chr16:65394334-65464602&hgt.customText=http://genomequebec.mcgill.ca/majewski/jas_UCSC/reg/3695303) |
| UBAP2 | 3203812 | core | rs10971752 | [3203812](http://genome.ucsc.edu/cgi-bin/hgTracks?org=human&db=hg17&position=chr9:33911744-34038937&hgt.customText=http://genomequebec.mcgill.ca/majewski/jas_UCSC/as/3203812) |
| TMEM77 | 2427753 | all | rs3762374 | [2427753](http://genome.ucsc.edu/cgi-bin/hgTracks?org=human&db=hg17&position=chr1:111371736-111395095&hgt.customText=http://genomequebec.mcgill.ca/majewski/jas_UCSC/reg/2427753) |
| PLD2 | 3707250 | core | rs3764897 | [3707250](http://genome.ucsc.edu/cgi-bin/hgTracks?org=human&db=hg17&position=chr17:4658059-4673670&hgt.customText=http://genomequebec.mcgill.ca/majewski/jas_UCSC/as/3707250) |
| MGC16169 | 2780811 | all | rs6843870 | [2780811](http://genome.ucsc.edu/cgi-bin/hgTracks?org=human&db=hg17&position=chr4:107284924-107597991&hgt.customText=http://genomequebec.mcgill.ca/majewski/jas_UCSC/reg/2780811) |
| MYO6 | 2914142 | core | rs2295936 | [2914142](http://genome.ucsc.edu/cgi-bin/hgTracks?org=human&db=hg17&position=chr6:76583942-76682621&hgt.customText=http://genomequebec.mcgill.ca/majewski/jas_UCSC/as/2914142) |
| USP8 | 3593685 | all | rs10220843 | [3593685](http://genome.ucsc.edu/cgi-bin/hgTracks?org=human&db=hg17&position=chr15:48503656-48580253&hgt.customText=http://genomequebec.mcgill.ca/majewski/jas_UCSC/reg/3593685) |
| ADD3 | 3263586 | all | rs4918478 | [3263586](http://genome.ucsc.edu/cgi-bin/hgTracks?org=human&db=hg17&position=chr10:111746152-111885408&hgt.customText=http://genomequebec.mcgill.ca/majewski/jas_UCSC/reg/3263586) |
| UEVLD | 3365492 | all | rs11024706 | [3365492](http://genome.ucsc.edu/cgi-bin/hgTracks?org=human&db=hg17&position=chr11:18507794-18567085&hgt.customText=http://genomequebec.mcgill.ca/majewski/jas_UCSC/reg/3365492) |
| CCDC41 | 3466174 | core | rs4761470 | [3466174](http://genome.ucsc.edu/cgi-bin/hgTracks?org=human&db=hg17&position=chr12:93204537-93331564&hgt.customText=http://genomequebec.mcgill.ca/majewski/jas_UCSC/as/3466174) |
| WARS | 3579582 | all | rs11622361 | [3579582](http://genome.ucsc.edu/cgi-bin/hgTracks?org=human&db=hg17&position=chr14:99869893-99912265&hgt.customText=http://genomequebec.mcgill.ca/majewski/jas_UCSC/reg/3579582) |
| CBFA2T3 | 3704592 | core | rs11648295 | [3704592](http://genome.ucsc.edu/cgi-bin/hgTracks?org=human&db=hg17&position=chr16:87468793-87570686&hgt.customText=http://genomequebec.mcgill.ca/majewski/jas_UCSC/as/3704592) |
| RSRC2 | 3475752 | all | rs1473553 | [3475752](http://genome.ucsc.edu/cgi-bin/hgTracks?org=human&db=hg17&position=chr12:121514090-121536384&hgt.customText=http://genomequebec.mcgill.ca/majewski/jas_UCSC/reg/3475752) |
| C8orf32 | 3114250 | all | rs13272492 | [3114250](http://genome.ucsc.edu/cgi-bin/hgTracks?org=human&db=hg17&position=chr8:124509348-124548593&hgt.customText=http://genomequebec.mcgill.ca/majewski/jas_UCSC/reg/3114250) |
| CCDC100 | 2873195 | all | rs1366337 | [2873195](http://genome.ucsc.edu/cgi-bin/hgTracks?org=human&db=hg17&position=chr5:122642056-122832561&hgt.customText=http://genomequebec.mcgill.ca/majewski/jas_UCSC/reg/2873195) |
| USP36 | 3772596 | core | rs3744802 | [3772596](http://genome.ucsc.edu/cgi-bin/hgTracks?org=human&db=hg17&position=chr17:74303994-74346413&hgt.customText=http://genomequebec.mcgill.ca/majewski/jas_UCSC/as/3772596) |
| WDR67 | 3114099 | core | rs7008277 | [3114099](http://genome.ucsc.edu/cgi-bin/hgTracks?org=human&db=hg17&position=chr8:124154191-124233530&hgt.customText=http://genomequebec.mcgill.ca/majewski/jas_UCSC/as/3114099) |
| TMEM149 | 3859924 | all | rs2293687 | [3859924](http://genome.ucsc.edu/cgi-bin/hgTracks?org=human&db=hg17&position=chr19:40924879-40928132&hgt.customText=http://genomequebec.mcgill.ca/majewski/jas_UCSC/reg/3859924) |
| IL6 | 2992594 | all | rs6949149 | [2992594](http://genome.ucsc.edu/cgi-bin/hgTracks?org=human&db=hg17&position=chr7:22513626-22544706&hgt.customText=http://genomequebec.mcgill.ca/majewski/jas_UCSC/reg/2992594) |
| DDX19A/B | 3667169 | core | rs7919 | [3667169](http://genome.ucsc.edu/cgi-bin/hgTracks?org=human&db=hg17&position=chr16:68881193-68964677&hgt.customText=http://genomequebec.mcgill.ca/majewski/jas_UCSC/as/3667169) |
| OVGP1 | 2427917 | core | rs1264888 | [2427917](http://genome.ucsc.edu/cgi-bin/hgTracks?org=human&db=hg17&position=chr1:111669007-111682414&hgt.customText=http://genomequebec.mcgill.ca/majewski/jas_UCSC/as/2427917) |
| BCKDHA | 3834195 | all | rs12602 | [3834195](http://genome.ucsc.edu/cgi-bin/hgTracks?org=human&db=hg17&position=chr19:46548787-46582459&hgt.customText=http://genomequebec.mcgill.ca/majewski/jas_UCSC/reg/3834195) |
| C17orf57 not valid | 3724617 | core | rs3760372 | [3724617](http://genome.ucsc.edu/cgi-bin/hgTracks?org=human&db=hg17&position=chr17:42756356-42873648&hgt.customText=http://genomequebec.mcgill.ca/majewski/jas_UCSC/as/3724617) |
| ATP5SL | 3863093 | core | rs1043413 | [3863093](http://genome.ucsc.edu/cgi-bin/hgTracks?org=human&db=hg17&position=chr19:46629348-46636116&hgt.customText=http://genomequebec.mcgill.ca/majewski/jas_UCSC/as/3863093) |
| ERAP2 | 2821389 | core | rs7716222 | [2821389](http://genome.ucsc.edu/cgi-bin/hgTracks?org=human&db=hg17&position=chr5:96241043-96279291&hgt.customText=http://genomequebec.mcgill.ca/majewski/jas_UCSC/as/2821389) |
| PARP2 | 3527423 | core | rs2297616 | [3527423](http://genome.ucsc.edu/cgi-bin/hgTracks?org=human&db=hg17&position=chr14:19881647-19895901&hgt.customText=http://genomequebec.mcgill.ca/majewski/jas_UCSC/as/3527423) |
| DKFZp451M2119 | 2588913 | core | rs4130304 | [2588913](http://genome.ucsc.edu/cgi-bin/hgTracks?org=human&db=hg17&position=chr2:177973838-178028867&hgt.customText=http://genomequebec.mcgill.ca/majewski/jas_UCSC/as/2588913) |
| USMG5 | 3304753 | core | rs7911488 | [3304753](http://genome.ucsc.edu/cgi-bin/hgTracks?org=human&db=hg17&position=chr10:105138821-105144095&hgt.customText=http://genomequebec.mcgill.ca/majewski/jas_UCSC/as/3304753) |

P-values in the core analysis have undergone FDR correction.
