# Supplementary material for: Fine-Scale Variation and Genetic Determinants of Alternative Splicing across Individuals
Source: PLoS Genet. 2009 Dec 11;5(12):e1000766. doi: 10.1371/journal.pgen.1000766 (PMC2780703; doi:10.1371/journal.pgen.1000766)
Supplement: Table S2 — SNPs discovered around AS events. (0.09 MB DOC) [file pgen.1000766.s003.doc]

Table S2 : SNPs discovered around AS events

| Gene and SNPs Track^1^ | Probeset |
| --- | --- |
| [ULK4](http://genome.ucsc.edu/cgi-bin/hgTracks?org=human&db=hg18&position=chr3:41931896-41933096&hgt.customText=http://genomequebec.mcgill.ca/majewski/jas_UCSC/allsnps/2670619) | 2670619 |
| [IL6](http://genome.ucsc.edu/cgi-bin/hgTracks?org=human&db=hg18&position=chr7:22733121-22734322&hgt.customText=http://genomequebec.mcgill.ca/majewski/jas_UCSC/allsnps/2992594) | 2992594 |
| [SF3B2](http://genome.ucsc.edu/cgi-bin/hgTracks?org=human&db=hg18&position=chr11:65581985-65583185&hgt.customText=http://genomequebec.mcgill.ca/majewski/jas_UCSC/allsnps/3335930) | 3335930 |
| [SNX11](http://genome.ucsc.edu/cgi-bin/hgTracks?org=human&db=hg18&position=chr17:43542501-43543701&hgt.customText=http://genomequebec.mcgill.ca/majewski/jas_UCSC/allsnps/3725089) | 3725089 |
| [ACP1](http://genome.ucsc.edu/cgi-bin/hgTracks?org=human&db=hg18&position=chr2:261616-262816&hgt.customText=http://genomequebec.mcgill.ca/majewski/jas_UCSC/allsnps/ACP1) | 2466156 |
| [APPBP1](http://genome.ucsc.edu/cgi-bin/hgTracks?org=human&db=hg18&position=chr16:65418844-65420045&hgt.customText=http://genomequebec.mcgill.ca/majewski/jas_UCSC/allsnps/APPBP1) | 3695303 |
| [ATP5SL](http://genome.ucsc.edu/cgi-bin/hgTracks?org=human&db=hg18&position=chr19:46630500-46631700&hgt.customText=http://genomequebec.mcgill.ca/majewski/jas_UCSC/allsnps/ATP5SL) | 3863093 |
| [AZIN1](http://genome.ucsc.edu/cgi-bin/hgTracks?org=human&db=hg18&position=chr8:103939934-103941134&hgt.customText=http://genomequebec.mcgill.ca/majewski/jas_UCSC/allsnps/AZIN1) | 3147621 |
| [C14orf129](http://genome.ucsc.edu/cgi-bin/hgTracks?org=human&db=hg18&position=chr14:95915262-95916462&hgt.customText=http://genomequebec.mcgill.ca/majewski/jas_UCSC/allsnps/C14orf129) | 3550335 |
| [SNORD49B](http://genome.ucsc.edu/cgi-bin/hgTracks?org=human&db=hg18&position=chr17:16283119-16284319&hgt.customText=http://genomequebec.mcgill.ca/majewski/jas_UCSC/allsnps/C17orf45andSNORD49B) | 3712109 |
| [AMACR](http://genome.ucsc.edu/cgi-bin/hgTracks?org=human&db=hg18&position=chr5:34039902-34041102&hgt.customText=http://genomequebec.mcgill.ca/majewski/jas_UCSC/allsnps/C1QTNF3andAMACR) | 2852757 |
| [VISA](http://genome.ucsc.edu/cgi-bin/hgTracks?org=human&db=hg18&position=chr20:3785841-3787041&hgt.customText=http://genomequebec.mcgill.ca/majewski/jas_UCSC/allsnps/C20orf29andVISA) | 3874507 |
| [C8orf32](http://genome.ucsc.edu/cgi-bin/hgTracks?org=human&db=hg18&position=chr8:124518999-124520200&hgt.customText=http://genomequebec.mcgill.ca/majewski/jas_UCSC/allsnps/C8orf32) | 3114250 |
| [C8orf59](http://genome.ucsc.edu/cgi-bin/hgTracks?org=human&db=hg18&position=chr8:86318151-86319352&hgt.customText=http://genomequebec.mcgill.ca/majewski/jas_UCSC/allsnps/C8orf59) | 3142947 |
| [CCDC41](http://genome.ucsc.edu/cgi-bin/hgTracks?org=human&db=hg18&position=chr12:93352604-93353805&hgt.customText=http://genomequebec.mcgill.ca/majewski/jas_UCSC/allsnps/CCDC41) | 3466174 |
| [CDK7](http://genome.ucsc.edu/cgi-bin/hgTracks?org=human&db=hg18&position=chr5:68581774-68582974&hgt.customText=http://genomequebec.mcgill.ca/majewski/jas_UCSC/allsnps/CDK7) | 2813489 |
| [DMKN](http://genome.ucsc.edu/cgi-bin/hgTracks?org=human&db=hg18&position=chr19:40689637-40690837&hgt.customText=http://genomequebec.mcgill.ca/majewski/jas_UCSC/allsnps/DMKN) | 3859789 |
| [DUSP18](http://genome.ucsc.edu/cgi-bin/hgTracks?org=human&db=hg18&position=chr22:29390418-29391619&hgt.customText=http://genomequebec.mcgill.ca/majewski/jas_UCSC/allsnps/DUSP18) | 3957502 |
| [ERAP2](http://genome.ucsc.edu/cgi-bin/hgTracks?org=human&db=hg18&position=chr5:96261091-96262291&hgt.customText=http://genomequebec.mcgill.ca/majewski/jas_UCSC/allsnps/ERAP2) | 2821389 |
| [ESPL1](http://genome.ucsc.edu/cgi-bin/hgTracks?org=human&db=hg18&position=chr12:51948328-51949528&hgt.customText=http://genomequebec.mcgill.ca/majewski/jas_UCSC/allsnps/ESPL1) | 3415861 |
| [FAM64A](http://genome.ucsc.edu/cgi-bin/hgTracks?org=human&db=hg18&position=chr17:6293670-6294871&hgt.customText=http://genomequebec.mcgill.ca/majewski/jas_UCSC/allsnps/FAM64A) | 3707965 |
| [HNRPH1](http://genome.ucsc.edu/cgi-bin/hgTracks?org=human&db=hg18&position=chr5:178975989-178977189&hgt.customText=http://genomequebec.mcgill.ca/majewski/jas_UCSC/allsnps/HNRPH1) | 2890160 |
| [IFI44L](http://genome.ucsc.edu/cgi-bin/hgTracks?org=human&db=hg18&position=chr1:78865883-78867084&hgt.customText=http://genomequebec.mcgill.ca/majewski/jas_UCSC/allsnps/IFI44L) | 2343481 |
| [MGC16169](http://genome.ucsc.edu/cgi-bin/hgTracks?org=human&db=hg18&position=chr4:107395008-107396208&hgt.customText=http://genomequebec.mcgill.ca/majewski/jas_UCSC/allsnps/MGC16169) | 2780811 |
| [MMAB](http://genome.ucsc.edu/cgi-bin/hgTracks?org=human&db=hg18&position=chr12:108482874-108484075&hgt.customText=http://genomequebec.mcgill.ca/majewski/jas_UCSC/allsnps/MMAB) | 3470844 |
| [MTMR12](http://genome.ucsc.edu/cgi-bin/hgTracks?org=human&db=hg18&position=chr5:32283034-32284235&hgt.customText=http://genomequebec.mcgill.ca/majewski/jas_UCSC/allsnps/MTMR12) | 2852298 |
| [PARP2](http://genome.ucsc.edu/cgi-bin/hgTracks?org=human&db=hg18&position=chr14:19882511-19883711&hgt.customText=http://genomequebec.mcgill.ca/majewski/jas_UCSC/allsnps/PARP2) | 3527423 |
| [PLD2](http://genome.ucsc.edu/cgi-bin/hgTracks?org=human&db=hg18&position=chr17:4669223-4670424&hgt.customText=http://genomequebec.mcgill.ca/majewski/jas_UCSC/allsnps/PLD2) | 3707250 |
| [PPIL2](http://genome.ucsc.edu/cgi-bin/hgTracks?org=human&db=hg18&position=chr22:20379418-20380619&hgt.customText=http://genomequebec.mcgill.ca/majewski/jas_UCSC/allsnps/PPIL2) | 3938300 |
| [RBCK1](http://genome.ucsc.edu/cgi-bin/hgTracks?org=human&db=hg18&position=chr20:338038-339239&hgt.customText=http://genomequebec.mcgill.ca/majewski/jas_UCSC/allsnps/RBCK1) | 3873192 |
| [RNASEN](http://genome.ucsc.edu/cgi-bin/hgTracks?org=human&db=hg18&position=chr5:31550790-31551991&hgt.customText=http://genomequebec.mcgill.ca/majewski/jas_UCSC/allsnps/RNASEN) | 2852054 |
| [SGOL1](http://genome.ucsc.edu/cgi-bin/hgTracks?org=human&db=hg18&position=chr3:20190923-20192123&hgt.customText=http://genomequebec.mcgill.ca/majewski/jas_UCSC/allsnps/SGOL1) | 2665585 |
| [SH3YL1](http://genome.ucsc.edu/cgi-bin/hgTracks?org=human&db=hg18&position=chr2:232242-233443&hgt.customText=http://genomequebec.mcgill.ca/majewski/jas_UCSC/allsnps/SH3YL1) | 2537134 |
| [SIDT1](http://genome.ucsc.edu/cgi-bin/hgTracks?org=human&db=hg18&position=chr3:114782333-114783533&hgt.customText=http://genomequebec.mcgill.ca/majewski/jas_UCSC/allsnps/SIDT1) | 2636499 |
| [TCP11](http://genome.ucsc.edu/cgi-bin/hgTracks?org=human&db=hg18&position=chr6:35211274-35212475&hgt.customText=http://genomequebec.mcgill.ca/majewski/jas_UCSC/allsnps/TCP11) | 2951389 |
| [TMEM77](http://genome.ucsc.edu/cgi-bin/hgTracks?org=human&db=hg18&position=chr1:111483087-111484287&hgt.customText=http://genomequebec.mcgill.ca/majewski/jas_UCSC/allsnps/TMEM77) | 2427753 |
| [SERGEF](http://genome.ucsc.edu/cgi-bin/hgTracks?org=human&db=hg18&position=chr11:17937048-17938249&hgt.customText=http://genomequebec.mcgill.ca/majewski/jas_UCSC/allsnps/TPH1andSERGEF) | 3365169 |
| [TMEM149](http://genome.ucsc.edu/cgi-bin/hgTracks?org=human&db=hg18&position=chr19:40925929-40927130&hgt.customText=http://genomequebec.mcgill.ca/majewski/jas_UCSC/allsnps/U2AF1L4andTMEM149) | 3859924 |
| [UBAP2](http://genome.ucsc.edu/cgi-bin/hgTracks?org=human&db=hg18&position=chr9:33962597-33963798&hgt.customText=http://genomequebec.mcgill.ca/majewski/jas_UCSC/allsnps/UBAP2) | 3203812 |
| [UEVLD](http://genome.ucsc.edu/cgi-bin/hgTracks?org=human&db=hg18&position=chr11:18511879-18513079&hgt.customText=http://genomequebec.mcgill.ca/majewski/jas_UCSC/allsnps/UEVLD) | 3365492 |
| [USMG5](http://genome.ucsc.edu/cgi-bin/hgTracks?org=human&db=hg18&position=chr10:105143438-105144638&hgt.customText=http://genomequebec.mcgill.ca/majewski/jas_UCSC/allsnps/USMG5) | 3304753 |
| [USP8](http://genome.ucsc.edu/cgi-bin/hgTracks?org=human&db=hg18&position=chr15:48563206-48564406&hgt.customText=http://genomequebec.mcgill.ca/majewski/jas_UCSC/allsnps/USP8) | 3593685 |
| [VEZT](http://genome.ucsc.edu/cgi-bin/hgTracks?org=human&db=hg18&position=chr12:94159499-94160700&hgt.customText=http://genomequebec.mcgill.ca/majewski/jas_UCSC/allsnps/VEZT) | 3426838 |
| [WARS](http://genome.ucsc.edu/cgi-bin/hgTracks?org=human&db=hg18&position=chr14:99909692-99910893&hgt.customText=http://genomequebec.mcgill.ca/majewski/jas_UCSC/allsnps/WARS) | 3579582 |
| [WDR67](http://genome.ucsc.edu/cgi-bin/hgTracks?org=human&db=hg18&position=chr8:124223169-124224369&hgt.customText=http://genomequebec.mcgill.ca/majewski/jas_UCSC/allsnps/WDR67) | 3114099 |
| [OVGP1](http://genome.ucsc.edu/cgi-bin/hgTracks?org=human&db=hg18&position=chr1:111770622-111771822&hgt.customText=http://genomequebec.mcgill.ca/majewski/jas_UCSC/allsnps/WDR77andOVGP1) | 2427917 |
| [ZNF419](http://genome.ucsc.edu/cgi-bin/hgTracks?org=human&db=hg18&position=chr19:62692748-62693949&hgt.customText=http://genomequebec.mcgill.ca/majewski/jas_UCSC/allsnps/ZNF419andZNF773) | 3843285 |
| [ERAP2](http://genome.ucsc.edu/cgi-bin/hgTracks?org=human&db=hg18&position=chr5:96263613-96264813&hgt.customText=http://genomequebec.mcgill.ca/majewski/jas_UCSC/allsnps/2821393) | 2821393 |

1: fold-change represent the average probeset expression fold change between homozygous sequenced individuals
